# Ketogenic diets as an adjuvant therapy in glioblastoma (the KEATING trial): study protocol for a randomised pilot study

**DOI:** 10.1186/s40814-017-0209-9

**Published:** 2017-11-28

**Authors:** Kirsty J. Martin-McGill, Anthony G. Marson, Catrin Tudur Smith, Michael D. Jenkinson

**Affiliations:** 1 Institute of Translational Medicine, University of Liverpool, Brownlow Hill, Liverpool, L69 3BX UK; 2 The Walton Centre NHS Foundation Trust, Lower Lane, Liverpool, L9 7LJ UK; 3 Department of Biostatistics, University of Liverpool, Brownlow Hill, Liverpool, L69 3BX UK

**Keywords:** Ketogenic diet, Modified ketogenic diet, Medium chain triglyceride diet, Glioblastoma

## Abstract

**Background:**

Glioblastoma is the commonest form of malignant brain tumour in adults, affecting 2–3 people per 100,000 per year. Despite current treatment options including surgical resection, radiotherapy and temozolomide chemotherapy, overall survival at 2 years is approximately 27%, with a median survival of 12–14 months. The ketogenic diet (KD) is postulated to work by simulating the metabolic response to fasting by promoting the utilisation of ketones as a primary energy source, and depriving the glycolytic pathways utilised by malignant glioma cells for growth. At present, there is no consensus as to which KD is preferable, with previous case series using different KDs, at different points in the treatment pathway. The aim of this randomised pilot study is to investigate protocol feasibility, tolerability and the impact on patient health and quality of life of two different KDs within an NHS setting. The results of this pilot study will inform which KD will be most deliverable and adhered to by patients in order to test for effectiveness in future trials.

**Methods:**

A prospective, non-blinded, randomised, pilot study will be undertaken in 12 patients with newly diagnosed glioblastoma treated by surgical resection. Patients will be randomised in a ratio of 1:1, using a permuted block randomisation method to one of two diets; the modified ketogenic diet and the medium chain triglyceride ketogenic diet. Primary data collection will take place 12 weeks after starting the diet and secondary data collection after 12 months. Feasibility will be assessed by retention and recruitment rates, ability to enrol patients prior to starting chemoradiotherapy, dietary compliance and adjustments, ketone levels, glucose levels and intervention time. Patient impact will be assessed through quality of life and food acceptability questionnaires, gastrointestinal side effects and changes to biochemical markers and anthropometric measures, assessed at regular intervals.

**Discussion:**

The results of this pilot study will be used to inform the feasibility, methodological design and power calculations of future phase III clinical trials investigating the effectiveness of KD as an adjuvant therapy in the management of glioblastoma.

**Trial registration:**

ISRCTN71665562 and NCT03075514.

## Background

Glioblastoma (GB) is the commonest form of malignant brain tumour in adults, affecting 2–3 people per 100,000 per year [1]. Despite current treatment options including maximal safe resection, radiotherapy and temozolomide chemotherapy [2], overall survival at 2 years remains poor (median survival of 12–14 months [3]). Several recent trials investigating newer chemotherapy agents (e.g. RTOG 0825–Bevacizumab trial [4]) and targeted therapies (e.g., CENTRIC–Celengitide trial [5]) have not resulted in any improvement to prognosis. Therefore, alternative treatment options are being explored and there is increasing clinical interest for investigating the ketogenic diet (KD) therapy as an adjuvant treatment for patients with GB.

KDs are high fat, low carbohydrate diets, resulting in the production of ketones as a primary energy source, depriving the glycolytic pathways utilised for growth by GBs. Laboratory studies of the effect of KDs in glioma mouse models have demonstrated increased survival [6], enhanced radiotherapy sensitivity [7], improved chemotherapy signalling [8] and reduced peritumoural oedema [9].

Recent studies investigating the use of the KD in humans have focused on the feasibility, safety and efficacy, in view of the fact that the diet may be considered unpalatable and unacceptable to patients with a limited life expectancy. Several small case studies [10–13] and one pilot study [14] have been reported.

Various forms of the KD have been developed over recent years to improve palatability and compliance, whilst maintaining efficacy within the diets primary evidence base of paediatric epilepsy [15, 16]. The two least restrictive KD are the modified ketogenic diet (MKD) and the medium chain triglyceride (MCT) KD, both of which are currently used for treating paediatric epilepsy within the National Health Service (NHS) and have been utilised in previous glioblastoma case series.

The MKD induces ketosis through encouraging a high fat and low carbohydrate intake, without limiting protein, fluid or energy intakes. There is no need for a fasting start or hospitalisation to commence the diet [17]. MKD is likely to be the most flexible and palatable KDs; therefore, may be more suitable for adults undergoing oncological treatments.

MCT KD was first described by Huttenlocher, Wilbourn and Signore [18] as a modification to the classic KD. It allows for the inclusion of larger portions of carbohydrate, thus, improving dietary tolerance and acceptability. A recent study by Martuscello et al. [19] investigating the use of MCT KD in GB animal models found slower tumour progression, increased survival, increased body weight and positive changes to serum lipids, in comparison to a standard KD and controls.

## Rationale

The effect of lifestyle factors (including diet) on tumours is one of the top 10 priority areas for research identified by the James Lind Priority Setting Partnership in the Neuro-Oncology community [20]. To enable an adequately powered randomised controlled trial to be undertaken, investigating the efficacy of the ketogenic diet in the therapeutic management of glioblastoma, this preliminary study is important to test protocol methodology and to explore potential impact on patient quality of life and health. At present, it is not known which KD, if any, holds promise for further investigation of effectiveness. Therefore, this study will directly compare two KD (MKD and MCT KD) to determine which diet is most deliverable and best adhered to by patients in order to test clinical effectiveness, as a primary outcome (overall survival) in a future definitive trial, within an NHS glioblastoma population. In a trial of effectiveness, a control group would be required (such as ‘healthy eating’) and directly compared to the ‘successful’ dietary arm from this pilot.

## Methods

### Aims and objectives

The aim of this trial is to investigate protocol feasibility and patient impact by comparing two KDs in an NHS setting, with a view to informing the design of future phase III clinical trials. The primary objective is to estimate retention rates to inform sample size calculations of future, definitive trials. Secondary objectives in relation to protocol feasibility include estimations of recruitment, enrolment and long-term retention rates and to obtain data on dietary compliance, dietary adjustments required to achieve ketosis, ketone and glucose levels, intervention time, protocol refinements and completeness of data. Secondary objectives related to patient impact include collating data on quality of life, food acceptability, gastrointestinal side effects, biochemical markers and anthropometry.

### Design

This is a prospective, non-blinded, randomised, pilot study which will be undertaken in patients diagnosed with newly diagnosed GB. Patients will be randomised to receive one of two types of KD; MKD or MCT KD. The trial has received ethical approval from the North West–Greater Manchester West Research Ethics Committee (17/NW/0013) and has been registered with the International Standard Randomised Controlled Trial Number registry (reference number 71665562) and ClinicalTrials.Gov (reference number NCT03075514).

### Setting

This single centre pilot trial will be conducted at The Walton Centre NHS Foundation Trust (WCFT), Liverpool, UK. WCFT is a dedicated neuroscience hospital.

### Participants

The target population is adults with newly, histologically diagnosed GB. All patients considered for the trial must meet the following inclusion criteria:

1. Age ≥ 16 years,
2. Patient at WCFT,
3. Performance status ≤ 2^1^ (numerical grading of patients well-being and function) [21],
4. Confirmed histological diagnosis of GB (WHO grade IV, [22]),
5. Undergone surgical resection and will go onto receive chemoradiotherapy with temozolomide.

Patients exhibiting the following will be excluded from the study:

1. Having any prior use of a KD,
2. Kidney dysfunction,
3. Liver dysfunction,
4. Gall bladder dysfunction,
5. Metabolic disorder,
6. Eating disorder (history of anorexia nervosa, bulimia nervosa, binge eating disorder),
7. Diabetes (requiring medication),
8. Body mass index (BMI) ≤ 18.5 kg/m^2^,
9. Use of weight loss medications,
10. Currently pregnant,
11. Performance status ≥ 3 [21].

### Sample size and recruitment

This study is being conducted as part of a PhD thesis. Twelve patients will be recruited over a 12 months period. Previous feasibility work shows this recruitment rate is achievable. Patients will be identified at a weekly neuro-oncology multi-disciplinary team (MDT) meeting; with documentation within MDT case sheet deemed an appropriate referral. Patients will be approached for recruitment after surgery and histological diagnosis of GB. Recruitment will take place for 12 months from the date of opening. Patients referred for the KEATING trial will be offered a screening appointment. A screening log will be maintained for all patients referred to the trial and reasons for ineligibility will be noted.

### Allocation strategy

Patients will be randomised into MKD or MCT KD groups. The patients will be informed of diet group by telephone. A permuted block randomisation method will be adopted, at a 1:1 ratio using ‘sealedenvelope’™ randomisation system. This will be set up and administered by the statistician (CTS) who is not involved with the recruiting of patients.

### Intervention design

**Fig. 1:**
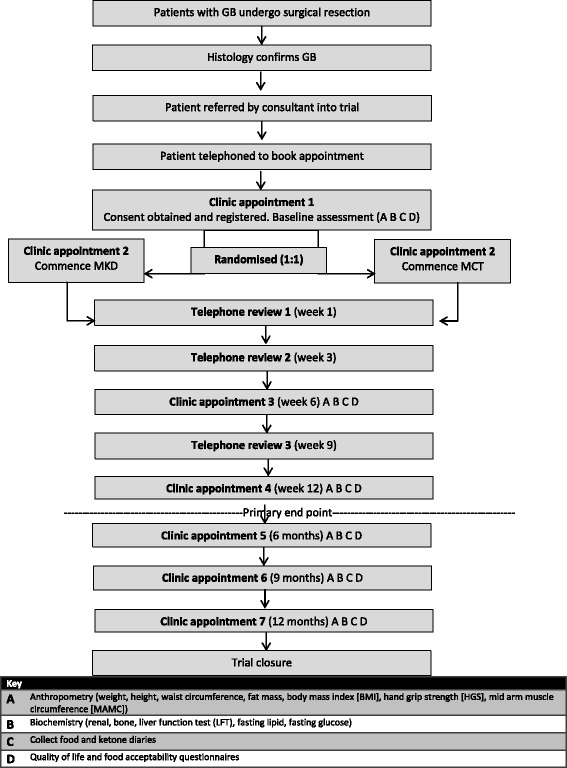
Flow of patients through the KEATING trial

All participants will be invited to attend clinical consultations pre-diet, on initiating diet, 6 weeks from initiation, 12 weeks from initiation (primary completion) and every 3 months for a total of 12 months (secondary completion) or until dietary discontinuation if prior to this. Telephone consultations will take place post initiation at weeks 1, 3 and 9 to enable dietary ‘fine tuning’ (Table 1 and Fig. 1). All consultations will be undertaken with the same research dietitian. It is not possible to blind the participants or the investigators due to differences between the diets and dietary education required.

**Table 1:** Study schedule

	Study period										
		Enrolment/diet allocation	Post allocation (on diet)								Close out
Timeline		1 week post-histology	1 week after clinic 1	1 weeks after clinic 2	3 weeks after clinic 2	6 weeks after clinic 2	9 weeks after clinic 2	12 weeks after clinic 2	6 months after clinic 2	9 months after clinic 2	12 months after clinic 2
Visit window	Post-histology	± 5 days	± 5 days before radiotherapy	± 3 days	± 3 days	± 5 days	± 3 days	± 5 days	± 10 days	± 10 days	± 10 days
Clinic visit	Telephone discussion	Clinic appointment 1 Consent, register, baseline assessment, randomisation	Clinic appointment 2 Commence diet	Telephone review 1	Telephone review 2	Clinic appointment 3 Diet review	Telephone review 3	Clinic appointment 4 Diet review	Clinic appointment 5 Diet review	Clinic appointment 6 Diet review	Clinic appointment 7 Diet review End of trial
Information sheet	X										
Informed consent		X									
Eligibility screen		X									
Randomisation		X									
Medical history review		X									
Medications review		X									
Anthropometry		X				X		X	X	X	X
Biochemistry		X						X	X	X	X
Food diary		X				X		X	X	X	X
Ketone diary						X		X	X	X	X
Quality of life (QoL) questionnaire EORCT QLQ C30		X				X		X	X	X	X
QoL questionnaire QLQ BN20		X				X		X	X	X	X
Food Acceptability Questionnaire		X				X		X	X	X	X
Dietary review		X	X	X	X	X	X	X	X	X	X
Ketone review				X	X	X	X	X	X	X	X

X = procedure carried out

### Dietary intervention

The dietary intervention will commence following surgery and prior to starting chemoradiotherapy. Oncology treatment will continue in line with the standard of care.

Both MKD and MCT KD are high in fat and low in carbohydrate. However, they contain different amounts and types of fat. The MKD provides fat to 80% of total energy requirements (predominately long-chain fatty acids) and 5% carbohydrate. Whilst the MCT KD provides fat to 75% of total energy requirements (30% of which is medium chain fatty acids, consumed via a nutritional supplement), and 10% carbohydrate. Protein is allowed freely in both diets.

Patients (and relatives when appropriate) will receive dietetic counselling and be provided with dietary literature to calculate intakes of these food groups. All patients will commence the diet at home, without a fasting start.

Energy requirements will be appropriate for patient’s age, weight, activity and metabolic stress, monitored through weight checks. To estimate energy requirements a 3-day weighed food diary will be analysed using DietPlan 7© (Forestfield Software LTD, Horsham, UK) and compared to the Parenteral and Enteral Nutrition Group (PENG) energy requirements [23–29], from which a mean energy requirement will be estimated. The requirements can be tailored for weight loss, gain or maintenance dependent upon the patient’s needs and wishes. Dietary requirements will be recalculated each time a new weight is obtained.

Seven-day diet plans will be calculated by the dietitian based upon total energy requirements. Nutritional content will be analysed using DietPlan 7© (Forestfield Software LTD, Horsham, UK) to ensure appropriate proportions of carbohydrate, fat and protein.

### Associated medications and treatments

Medications permitted: all medications with the exception of those specified below.

Medications not permitted: weight loss medications (including Orlistat, Belviq, Contrave, Saxenda, Phentermine and Qsymia) and any agents used in diabetic therapy (including biguanides, sulfonylureas, meglitinide derivatives, alpha-glucosidase inhibitors, thiazolidinediones (TZDs), glucagon-like peptide–1 (GLP-1) agonists, dipeptidyl peptidase IV (DPP-4) inhibitors, selective sodium-glucose transporter-2 (SGLT-2) inhibitors, insulins, amylinomimetics, bile acid sequestrants and dopamine agonists).

Data will be collected on use of corticosteroids, antiemetics, laxatives and antiepileptic drugs. Data will also be collected for nutritional supplements consumed by the patient.

### Baseline data collection

Baseline data will be collected at the initial consultation once eligibility is confirmed and written consent obtained by the investigator. Baseline data will include past medical history, symptoms at presentation, tumour location, surgical procedure, histopathology and molecular pathology subtypes for glioblastoma (MGMT, IDH-1, ATRX, 1p/19q status), current medication (prescribed and purchased), current nutritional supplementation (vitamins, minerals, herbal and nutritional products), food allergies or intolerances, level of physical activity (as defined by DH [25]), food or fluid texture modification, use of enteral feeding tube, anthropometry (height, weight, BMI, mid-arm muscle circumference, triceps skinfold, mid-arm muscle circumference, hand grip strength, waist circumference and fat mass), biochemistry (fasting lipid, fasting glucose, liver, renal and bone profiles), habitual 3 day weighed food diary, habitual gastrointestinal complaints, quality of life measures (EORTC QLQ C30 and QLQ BN 20 questionnaires) and habitual food acceptability (Food Acceptability Questionnaire, [30]).

Histopathology and molecular pathology subtypes for glioblastoma (MGMT, IDH-1, ATRX, 1p/19q status) classification will be conducted as per the current standard of care.

MRI will be conducted as per the current standard of care for patients with GB [31], resulting in scans at the following intervals:

- Pre-surgery
- Post-surgery (within 72 h)
- Pre-radiotherapy
- 1 month post-radiotherapy
- Mid chemotherapy (after 3 cycles)
- Post-chemotherapy (after 6 cycles)
- Every 6 months from 12 to 24 months

MRI scans will include T1 ± gadolinium, T2 and fluid-attenuated inversion recovery (FLAIR) sequences. Extent of resection on post-operative MRI will be determined, and recurrence will be measured according to the Response Assessment in Neuro-Oncology (RANO) criteria [32].

Changes to medications and treatment will be recorded; these are permitted to be altered in-line with the treating oncologist/neurosurgeons recommendations. Details of concomitant radiotherapy and chemotherapy and adjuvant chemotherapy will be noted in the CRF.

### Primary outcome measures

1. Assess retention and drop-out rates defined as:
  - The number of patients who start randomised treatment as a proportion of the number randomised, with reasons for non-compliance.
  - The number of patients who complete 12 weeks of diet as a proportion of the number randomised, with reasons for non-compliance.
  - The time to dietary discontinuation (week 12 or duration to discontinuation if prior to this).
  - A description of barriers and facilitators to data collection and participant retention will also be included.

### Secondary outcome measures

1. Estimation of recruitment rates
  - Actual recruitment rates will be compared to the proposed recruitment figures (12 patients over 12 months) with purpose of demonstrating trial feasibility for future, potential phase III clinical trials.
2. Enrolment of patients
  - Ability to comply with protocol enrolment timelines will be assessed by the number of patients initiated on diet prior to starting chemoradiation treatment. This will inform the feasible timelines in future clinical trials.
3. Long term retention
  - The time to dietary discontinuation after week 12.
4. Dietary adjustments required to achieve ketosis
  - Dietary adjustments advised by the dietitian will be recorded in the case report form (CRF) to inform the macronutrient composition of MKD and MCT KD required to achieve ketosis in this population to inform future protocols.
5. Dietary compliance
  - Dietary compliance will be self-reported by the patient at clinic appointments. The dietitian will also analyse self-reported 3-day weighed food diaries (completed at weeks 6, 12 and every 3 months thereafter) using DietPlan 7© (nutritional analysis computer programme). The results will be compared to dietary fat and carbohydrate requirements (calculated at previous clinic appointment) and percentage compliance rates will be calculated.
6. MCT compliance
  - MCT supplement compliance will be self-reported through 3-day weighed food diaries collected at week 6, week 12 and every 3 months thereafter. The results will be compared to the MCT dose advised (at previous clinic appointment) and a percentage compliance rate will be calculated.
7. Ketone and glucose levels
  - Ketosis will be monitored by patients self-reporting urinary ketone levels twice per day for the first 6 weeks then then once per week thereafter. Adequate urinary ketosis is defined as ≥ 4 mmol/L.
  - Blood ketones and blood glucose levels will be monitored weekly. Adequate blood glucose levels are defined as 3–5 mmol/L.
  - There are no robust guidelines for adequate levels of blood ketosis in adults with GB, however, from preliminary work by Meidenbauer et al. [33], levels of 2–4 mmol/L to be beneficial, therefore patients will be asked to record levels to aid future research.
  - All figures will be recorded in the ketone/glucose diary provided. The dietitian will assess these at each point of contact.
8. Dietetic time required for the interventions
  - Dietetic time spent on both clinical and non-clinical activities related to the trial will be recorded to aid future protocol design.
9. Protocol refinements required
  - Deviations from the protocol will be documented on the deviation log. This will be used to refine future protocols and inform future clinical trials.
10. Sample size estimates for future trials
  - Data synthesised from this pilot will inform sample size calculations for future phase III clinical trials based on the primary outcome measure of retention.
11. Completeness of data for all trial outcomes
  - Completeness of documented data will be assessed to inform feasibility of future clinical trials.
12. Quality of life
  - Quality of life will be evaluated though the generic EORTC QLQ C30 and brain cancer-specific QLQ BN 20 validated questionnaires prior to commencing the diet, at week 6, week 12 and every 3 months thereafter or at point of dietary discontinuation if prior to this.
13. Food acceptability
  - Food acceptability will be assessed through the non-validated Food Acceptability Questionnaire [30] completed prior to commencing the diet, at week 6, week 12 and every 3 months thereafter or at point of dietary discontinuation if prior to this.
14. Gastrointestinal side effects
  - Gastrointestinal side effects will be quantified from the EORCT QLQ C30 questionnaire and through informal clinic assessments. The Common Terminology Criteria for Adverse Events ([CTCAE], version 4.0) will be used to grade gastrointestinal side effects.
15. Changes to biochemical markers
  - Biochemical markers (fasting lipid, fasting glucose, liver, renal and bone profiles) will be undertaken at baseline and repeated every 3 months until discontinuation of diet.
16. Anthropometric changes
  - Anthropometry (height, weight, body mass index (BMI), waist circumference, mid-upper arm circumference (MUAC), mid-arm muscle circumference (MAMC), tricep skinfold (TSF), hand grip strength (HGS) and fat mass) will be measured at baseline, week 6, week 12 and every 3 months thereafter. All measurements will be undertaken as per measurement methodology cited in Parenteral and Enteral Nutrition Group (PENG), pocket guide to clinical nutrition [29].

### Withdrawal

If there is a change in the patient’s condition that in the clinician’s opinion justifies dietary discontinuation or if the patient withdraws consent, the patient will be withdrawn from dietary treatment. In this case, data up until the point of withdrawal will be used in analysis. Unless the patient specifically withdraws consent further data will be gathered for progression-free survival and overall survival analysis.

Patients will be made aware at the point of consent that they can withdraw at any time. No reason will be required. Should a patient withdraw, data will be included in the analysis until the point of withdrawal, unless the patient states otherwise. If they wish for their data to be excluded entirely, a CRF for destruction of data will be completed.

### Defining success

Pilot success will be graded using a traffic light system. The pilot will be deemed a success and progression to phase III trial will be considered appropriate if the following criteria are met:

- Green (go):
  - Recruitment rate of ≥ 75% of target (*n* = 9) achieved within the 12-month recruitment period.
  - ≥ 75% of patients commenced KD prior to chemoradiotherapy.
  - Retention rate of ≥ 75% at 3 months.
  - Diet acceptable to ≥ 75% of patients at 3 months.
  - ≥ 75% of the proposed data collection completed for each end point.
- Amber (review):
  - Recruitment rate of ≥ 50% of target (*n* = 6) achieved within the 12-month recruitment period.
  - ≥ 50% of patients commenced KD prior to chemoradiotherapy.
  - Retention rate of ≥ 50% at 3 months.
  - Diet acceptable to ≥ 50% of patients at 3 months.
  - ≥ 50% of the proposed data collection completed for each end point.
- Red (stop):
  - Recruitment rate < 50% of target (*n* = 5) achieved within the 12-month recruitment period.
  - < 50% of patients commenced KD prior to chemoradiotherapy.
  - Retention rate of < 50% at 3 months.
  - Diet acceptable to < 50% of patients at 3 months.
  - < 75% of the proposed data collection completed for each end point.

Retention rate, dietary acceptance and data collection will be assessed for each diet independently. Recruitment rate and dietary commencement will be assessed using data combined from both arms.

Any components of the pilot considered not feasible or unacceptable to patients will be evaluated prior to progression onto phase III clinical trial is considered. The Shanyinde, Pickering, and Weatherall [34] method will be used to categorise and assess the extent of the issue and the Bugge et al. [35] method will be used to evidence the decision-making process.

### Statistical considerations

#### Sample size

Feasibility data demonstrates a likely retention rate of 70% participants at 12 weeks. With a sample size of 12, we will be able to estimate retention rates of 70% to within a 95% confidence interval of ± 25.93% (Hooper [36]). Billingham et al. [37] demonstrate a median sample size of 30 (range 8–114 participants) for UK pilot studies, whilst Hertzog [38] reports on the statistical adequacy for sample sizes of 10–40, thus further justifying a sample size of 12 for the current trial.

#### Analysis plan

A detailed analysis plan will be developed prior to the final analysis. In brief, descriptive statistics will be used to summarise retention, recruitment rates, enrolment adherence, dietary adjustments, dietetic time, dietary compliance, MCT supplement compliance, ketosis, anthropometry changes, biochemistry changes, quality of life, food acceptability and gastrointestinal side effects. The Kaplan-Meier survival curve will be estimated for overall survival and progression-free survival and displayed graphically with 95% confidence intervals.

## Discussion

### Practical considerations

When designing the trial, we considered a number of practical issues to enable implementation within an NHS setting.

When deciding on which KDs to include within the trial, nutritional adequacy and previous evidence base were considered. There are various types of KDs, offering varying contents of fat and carbohydrate. From the literature the two KDs previous cited in oncology case series include the MKD and the MCT KD [13, 14, 39, 40]. Both diets allow for the sufficient provision of protein, essential for patients undergoing oncological treatments, in comparison to the classic ketogenic diet which would not meet adult protein requirements. Both MCT and MKD are currently used in NHS practice for patients with refractory epilepsy.

In this trial, the KD will be offered alongside current standard of care. In order to allow for future meta-analysis of data, the diet will be commenced post-surgical resection, prior to chemoradiotherapy in keeping with US centres [41, 42]. This theory is supported by previous animal models which illustrate the KD to enhance the effects of chemoradiation [6–8].

Methods for assessing ketone and glucose levels were also considered. Blood ketone and glucose levels can provide a more accurate result, when compared to urinary ketones, due to the effects of urinary dilution. However, blood monitoring is more invasive for the patient and considerably more expensive when considering trial design and potential future NHS implementation. The minimum ketone monitoring requirements cited by the International League Against Epilepsy Task Force for Diet Therapy state urinary ketones to be a minimum standard [43]. Therefore, in keeping with this, we will conduct urinary ketone monitoring, with additional weekly blood ketone and glucose levels (home finger prick testing), with a view to informing future methodology.

In terms of recruitment, 12 patients is an achievable target and suitable for a pilot study, however, an element of statistical uncertainty will be present. Therefore, we have opted for descriptive statistical methods and will apply caution when interpreting results for any future study design.

### Data collection and storage

Data will be pseudo anonymised and transferred onto a password protected electronic spreadsheet (Microsoft Excel©) held on University of Liverpool Active DataStore.

Data will be held for 10 years to allow for future retrospective comparisons to larger scale studies. After the trial is complete, the essential trial paper documentation and CRFs will be archived by the University of Liverpool Records Management Department and held at University Records Centre. Electronic data will be archived by the investigator, in the University of Liverpool Data Catalogue, as per the University of Liverpool, Research Data Management Policy. Source documents are held within the medical notes, therefore are retained in the Health Records Library of WCFT, as per the WCFT Clinical Records Management Policy.

### Patient and public involvement

A PPI event was undertaken, seeking the active involvement of patients and public in identifying research priorities and outcome measures for KD in GB. Following feedback from the event improvements were made to patient information leaflets, plain language summary, recruitment processes, clinical consultations, along with reducing patient costs to enhance trial participation.

### Adverse events

The main health risks associated with the diet include gastrointestinal intolerance (such as diarrhoea, constipation, nausea, reflux, abdominal discomfort), altered or raised cholesterol, kidney stones and decreased bone density [44].

To monitor these health risks biochemistry will be monitored every 3 months (renal, bone, LFT, fasting cholesterol and glucose). Gastrointestinal side effects will be assessed using the Common Terminology Criteria for Adverse Events (CTCAE, version 4, [45]), and dietary adjustments will be made to aid symptom relief when possible.

All adverse events (AE) will be reported. The National Institute of Health Research (NIHR) ‘decision tree for adverse event reporting’ [46] will be used to grade AE and serious adverse events (SAE) severity.

An AE or SAE will be reported by the investigator; however the chief investigator will be responsible for determining causality. The trial would close early should SAEs occur as a direct result of the trial intervention.

Trigger monitoring will be adopted which will be managed by the University of Liverpool (the Sponsor).

All SAE will be reported to the Trial Steering Committee within 1 week of the event.

## Conclusion

This pilot study will be the first in the UK to investigate the feasibility and tolerability of KD in GB patients. By testing protocol feasibility, we will determine which diet is most deliverable and adhered to by patients, and this will inform the methodology of future phase III clinical trials, from which effectiveness could be determined. We will also generate data on diet acceptability and the impact on patient quality of life and anthropometry.

## Trial status

Current protocol version 1, 13 December 2016. Recruitment began 1 April 2017. Recruitment expected to be completed by 31 March 2018.

## Abbreviations

AE: Adverse event
ATRX: Alpha-thalassemia/mental retardation syndrome x-linked mutation
BMI: Body mass index
CI: Chief investigator
CRF: Case report form
EORTC: European Organisation for Research and Treatment of Cancer
FLAIR: Fluid-attenuated inversion recovery
GB: Glioblastoma
IDH-1: Isocitrate dehydrogenase 1
KD: Ketogenic diet
MCT KD: Medium chain triglyceride ketogenic diet
MDT: Multi-disciplinary team
MGMT: O^6^-methylguanin-DNA-methyltransferase
MKD: Modified ketogenic diet
MRI: Magnetic resonance imaging
NHS: National Health Service
PENG: Parenteral and Enteral Nutrition Group
PPI: Patient and public involvement
QLQ: Quality of life questionnaire
RANO: Response Assessment in Neuro-Oncology
SAE: Serious adverse event
UK: United Kingdom
WCFT: Walton Centre NHS Foundation Trust

## Footnotes

1 Performance status (PS) 0 means normal activity; PS 1 means some symptoms but nearly fully ambulant; PS 2 means < 50% daytime in bed; PS 3 means > 50% daytime in bed; PS 4 is completely bed bound [21].
